# Association between prophylactic antibiotics and surgical site infections in bimaxillary orthognathic surgery: a retrospective study

**DOI:** 10.1186/s12903-025-07246-x

**Published:** 2025-11-24

**Authors:** Ryuta Urakawa, Yuto Horie, Minako Ohishi, Yasuko Machida, Yoshiko Ikeda, Hiromi Nagashima, Fumie Toda, Hiroko Ueda, Kazunori Nozaki, Shiho Mima, Soju Seki, Yusuke Yokota, Emiko Tanaka Isomura, Kenji Ikeda

**Affiliations:** 1 Department of Pharmacy, The University of Osaka Dental Hospital, 1-8 Yamada-oka, Suita, Osaka 565-0871 Japan; 2 Department of Clinical Pharmacy Research and Education, Graduate School of Pharmaceutical Sciences, The University of Osaka, 1-6 Yamada- oka, Suita, Osaka 565-0871 Japan; 3 Department of Clinical Pharmacy Research and Education, School of Pharmaceutical Sciences, The University of Osaka, 1-6 Yamada-oka, Suita, Osaka 565-0871 Japan; 4 Division for Oral Dental Informatics, The University of Osaka Dental Hospital, 1-8 Yamada-oka, Suita, Osaka 565-0871 Japan; 5 Department of Oral and Maxillofacial Surgery, Graduate School of Dentistry, The University of Osaka, 1-8 Yamada-oka, Suita, Osaka 565- 0871 Japan

**Keywords:** Antimicrobial prophylaxis, Bimaxillary osteotomy, Postoperative infection, Antimicrobial resistance, Surgical site infection

## Abstract

**Background:**

Surgical site infections (SSIs) are a common complication after bimaxillary orthognathic surgery. While sulbactam/ampicillin and cefmetazole are recommended in Japan, alternatives such as ampicillin and clindamycin are also used, with limited comparative evidence. This study assessed the impact of these antibiotics on SSI incidence and related risk factors.

**Methods:**

We retrospectively analyzed 115 patients who underwent Le Fort I and bilateral sagittal split osteotomy at the University of Osaka Dental Hospital between January 1 and December 31 in 2023. Patients were categorized into four groups based on the prophylactic antibiotic administered: sulbactam/ampicillin (*n* = 70), ampicillin (*n* = 8), cefmetazole (*n* = 25), and clindamycin (*n* = 12). The duration of antibiotic administration was standardized within each group. Clinical variables such as age, sex, body mass index, third molar extraction, temporary anchorage devices insertion, operative time, and estimated blood loss were collected and compared between groups. SSI was defined as a postoperative infection requiring antibiotic treatment occurring within 30 days (or within 1 year if implants were used).

**Results:**

There were no significant differences in patient characteristics among the four antibiotic groups. SSI incidence was significantly higher in the ampicillin group compared to the sulbactam/ampicillin group (*p* = 0.046), while no other pairwise comparisons among the groups revealed statistically significant differences. Other clinical variables were not significantly associated with SSI occurrence.

**Conclusion:**

Ampicillin monotherapy may be insufficient for preventing SSIs in bimaxillary orthognathic surgery. Sulbactam/ampicillin demonstrated superior efficacy, whereas cefmetazole and clindamycin showed comparable effectiveness as alternative prophylactic agents. These findings underscore the importance of selecting appropriate antibiotics to minimize postoperative infection risk.

## Background

Orthognathic surgery is the standard treatment for dentofacial deformities and malocclusion due to skeletal discrepancies. To correct the skeletal positions of the maxilla and mandible, Le Fort I osteotomy and bilateral sagittal split osteotomy (BSSO) are commonly employed as the primary surgical techniques [1, 2]. Surgical site infection (SSI) is one of the major complications associated with orthognathic surgery, with reported incidence rates ranging from 1.4% to 33.4%[3]. SSIs can lead to patient discomfort, prolonged hospitalization, increased postoperative morbidity and mortality, and higher healthcare costs. Therefore, preventing SSIs is essential to achieving favorable clinical outcomes in patients undergoing orthognathic surgery [4–6]. 

The Japanese Clinical Practice Guidelines for antimicrobial prophylaxis in surgery recommend sulbactam/ampicillin or cefmetazole as the first-line prophylactic antibiotics for preventing surgical infections. For patients with β-lactam allergies, clindamycin is recommended as the first choice [7, 8]. This guideline recommends administering prophylactic antibiotics for no more than 48 h postoperatively; however, a Japanese report suggests continuing antibiotics for more than 4 days [9]. From an antimicrobial stewardship perspective, the use of narrow-spectrum antibiotics that target likely pathogens, for the shortest possible duration, is strongly encouraged. However, evidence regarding the optimal choice of prophylactic antibiotics specifically for infection prevention in orthognathic surgery remains limited[10, 11].

In Japan, although the use of sulbactam/ampicillin and cefmetazole is recommended by clinical guidelines for orthognathic surgery, these drugs are not covered by national health insurance for perioperative prophylaxis in orthognathic surgery. Additionally, due to global antibiotic supply shortages, Japan has experienced a significant decline in the availability of these medications, necessitating the temporary use of alternative antibiotics. At our institution, due to the limited availability of sulbactam/ampicillin and cefmetazole, alternative antibiotics such as ampicillin and clindamycin were used during certain periods.

In this retrospective study, we aimed to assess the incidence of SSIs in bimaxillary orthognathic surgery under different prophylactic antibiotic regimens and identify potential risk factors. Through this investigation, we sought to determine the most effective prophylactic antibiotic regimen for SSI prevention in orthognathic procedures.

## Materials and methods

### Study population

This study included patients who underwent bimaxillary osteotomy (Le Fort I + BSSO) at The University of Osaka Dental Hospital between January 1 and December 31, 2023. Exclusion criteria were: (1) patients whose medical records did not cover the full postoperative observation period required for SSI diagnosis (i.e., 30 days for non-implant cases or 1 year for implant cases), and (2) patients who had a change in prophylactic antibiotics during the perioperative period.

### Data collection

Patient data were extracted from electronic medical records, including age, sex, body mass index (BMI), intraoperative blood loss, operative time, operating maxillofacial surgeon, placement of temporary anchorage devices (TADs), intraoperative third molar extraction, occurrence of SSI, and type and dosage of prophylactic antibiotics. SSIs were defined based on the Centers for Disease Control and Prevention (CDC) guidelines:[12] for procedures without implants, infections occurring within 30 days postoperatively in either or both jaws that required additional systemic antibiotic therapy due to clinical signs of infection (e.g., swelling, redness, pus discharge, or fever) were considered SSIs; for procedures with implant, infections occurring within 1 year were included. Supportive treatment such as NSAIDs or chlorhexidine mouthwash alone was not regarded as SSI. Patients were categorized into four groups by prophylactic antibiotic: sulbactam/ampicillin, ampicillin, cefmetazole, or clindamycin.

### Statistical analysis

Baseline characteristics between SSI and without SSI groups were compared using Fisher’s exact test for categorical variables and the Wilcoxon rank-sum test for continuous variables. The Kruskal–Wallis test assessed baseline differences in patient characteristics across the four antibiotic groups. Differences in the incidence of SSIs among the four operating maxillofacial surgeons and among the four antibiotic groups were also analyzed using Fisher’s exact test. Additionally, the Wilcoxon rank-sum test was used to compare SSI incidence rates among the antibiotic groups. All statistical analyses were performed using JMP version 17.1.0. (SAS Institute Inc., Cary, NC, USA) A two-sided p-value of less than 0.05 was considered statistically significant.

### Ethical considerations

This study was approved by the Ethics Committees of both The University of Osaka Dental Hospital, and Graduate School of Pharmaceutical Sciences, The University of Osaka (approval numbers R5-E29-2, Yakuhito202317).

## Results

Between January 1 and December 31, 2023, 131 patients underwent bimaxillary orthognathic surgery (Le Fort I osteotomy and bilateral sagittal split osteotomy). Sixteen patients were excluded: 2 due to insufficient follow-up records for SSI diagnosis and 14 due to changes in prophylactic antibiotic regimens during the perioperative period. As a result, 115 patients were included in the final analysis. Furthermore, none of these patients had generally considered risk factors for SSI, such as smoking, diabetes or steroid use, although data on oral hygiene was not systematically collected.

Patient demographics and clinical characteristics are summarized in Table 1. In the sulbactam/ampicillin group (*n* = 70), ampicillin group (*n* = 8), and cefmetazole group (*n* = 25), all patients received prophylactic antibiotics preoperatively and continued for 2 postoperative days. In the clindamycin group (*n* = 12), antibiotics were administered preoperatively and continued for 4 postoperative days.

**Table 1:** Background of study participants

Characteristics	*n* = 115
Mean Age (SD)	26.4 (7.9)
Gender	
Male: n (%)	39 (33.9)
Female: n (%)	76 (66.1)
Mean BMI (SD)	21.5 (3.0)
Median Operative time: min (range)	234 (158–419)
Median Amount of blood loss: mL (range)	240 (65–1125)
TAD placement: n (%)	20 (17.4)
Third molar extraction: n (%)	5 (0.04)
SSI onset: n (%)	23 (20.0)
Mandible: n	22
Maxilla: n	1
Prophylactic antibiotics	
SBT/ABPC: n (%)	70 (60.9)
ABPC: n (%)	8 (7.0)
CMZ: n (%)	25 (21.7)
CLDM: n (%)	12 (10.4)

*SD* Standard deviation, *BMI* Body mass index, *TAD* Temporary anchorage device, *SSI* Surgical site infection, *SBT/ABPC* Sulbactam/ampicillin, *ABPC* Ampicillin, *CMZ* Cefmetazole, *CLDM* Clindamycin

There were no statistically significant differences in the baseline characteristics among the four antibiotic groups (Table 2). Similarly, no patient background factors were significantly associated with SSI occurrence between the SSI and without SSI groups according to the Wilcoxon rank-sum test and Fisher’s exact test (Table 3).

**Table 2:** Comparison of patients between antibiotic prophylaxis groups

	SBT/ABPC (*n* = 70)	ABPC (*n* = 8)	CMZ (*n* = 25)	CLDM (*n* = 12)	*P* value
Mean age (SD)	26.5 (8.4)	28.9 (6.3)	24.7 (6.6)	27.7 (7.7)	0.11
Male/Female: n (%)	26 (37.1)/44 (62.9)	2 (25.0)/6 (75.0)	5 (20.0)/20 (80.0)	6 (50.0)/6 (50.0)	0.26
Mean BMI (SD)	21.6 (3.0)	21.2 (2.4)	21.1 (3.2)	21.8 (3.4)	0.68
Median operative time: min (range)	241.5 (174–419)	229.5 (178–271)	224 (158–312)	236 (191–346)	0.16
Median amount of blood loss: mL (range)	240 (65–915)	300 (85–470)	200 (90–780)	285 (165–1125)	0.49
TAD placement: n (%)	9 (12.9)	3 (37.5)	7 (28.0)	1 (8.3)	0.11
Third molar extraction: n (%)	2 (2.9)	0 (0)	2 (8.0)	1 (8.3)	0.41

Kruskal-Wallis test was performed for age, BMI, operative time, amount of blood loss. Fisher’s exact probability test was performed for gender, TAD placement, third molar extraction

**Table 3:** Comparison between patients with or without SSI (surgical site infection)

	SSI (*n* = 23)	Without SSI (*n* = 92)	*P* value
Mean age (SD)	27.0 (6.2)	26.2 (8.2)	0.16
Male/Female: n (%)	10 (43.5)/13 (56.5)	29 (31.5)/63 (68.5)	0.32
Mean BMI (SD)	22.3 (3.7)	21.3 (2.8)	0.28
Median operative time: min (range)	246 (178–364)	231 (158–419)	0.52
Median amount of blood loss: mL (range)	330 (85–595)	230 (65–1125)	0.18
TAD placement: n (%)	7 (30.4)	13 (14.1)	0.12
Third molar extraction: n (%)	1 (4.4)	4 (4.4)	1.00

Wilcoxon signed-rank test or Fisher’s exact probability test were performed

There were no statistically significant differences in SSI rates among the operating maxillofacial surgeons (Table 4). A statistically significant difference in SSI incidence was observed among the antibiotic groups (*p* = 0.03, Fisher’s exact test) (Table 5). Further pairwise comparison indicated a significant difference between the sulbactam/ampicillin group and the ampicillin group (*p* = 0.0077). Among the 23 patients who developed SSIs, bacterial cultures were performed in 6 cases, all of which predominantly yielded α-streptococci. In the remaining 17 patients, no cultures were obtained because there was no abscess formation or purulent discharge at the infection sites.

**Table 4:** Impact of the primary dental surgeon on SSI

Primary dental surgeon	SSI (*n* = 23)	Without SSI (*n* = 92)	*P* value
A: n (%)	7 (21.2)	26 (78.8)	0.84
B: n (%)	7 (22.6)	24 (77.4)	
C: n (%)	6 (21.4)	22 (78.6)	
D: n (%)	3 (13.0)	20 (87.0)	

Fisher’s exact probability test was performed. All orthognathic surgeries were performed by four oral and maxillofacial surgeons (A: 17 years, B: 13 years, C: 21 years, and D: 11 years of surgical experience). No trainees or junior surgeons were the primary operators in any cases

**Table 5.**
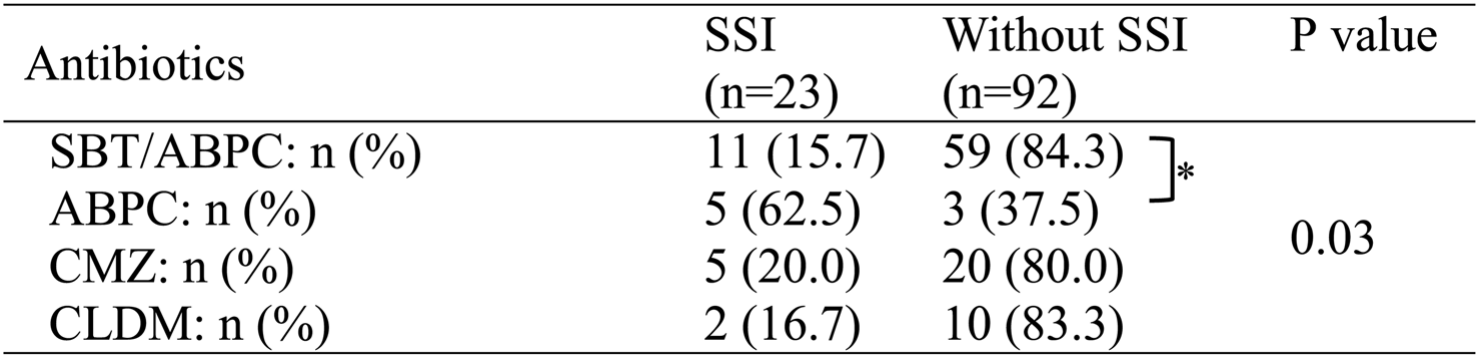
Impact of antimicrobial prophylaxis on SSI

Fisher’s exact test was used for overall comparison among the four groups. Subsequently, pairwise comparisons were performed with Bonferroni correction (adjusted significance threshold: *P* < 0.0083). An asterisk (*) indicates a statistically significant difference

## Discussion

In this study, we investigated the relationship between prophylactic antibiotics and the incidence of SSI in orthognathic surgery. The results shown in Tables 2 and 3, and 4 indicated that patient background factors, including TAD insertion, intraoperative third molar extraction, and differences among operating maxillofacial surgeons, were not significantly associated with the occurrence of SSIs. In contrast, Table 5 revealed a significantly higher SSI incidence in the ampicillin group compared to the sulbactam/ampicillin group (*p* = 0.0077), suggesting that combining β-lactamase inhibitors with penicillin antibiotics may be necessary to effectively prevent SSI. Furthermore, since no significant difference in SSI incidence was observed between the sulbactam/ampicillin group and either the cefmetazole group or the clindamycin group, these antibiotics may serve as viable alternatives to sulbactam/ampicillin.

Several factors have been reported as potential contributors to SSIs in orthognathic surgery, including age, sex, operative time, third molar extraction, and perioperative antibiotic use. One study suggested that older patients are at higher risk[13], while another has found no significant association [14]. Similarly, male sex has been reported as a risk factor in a study[9], whereas another found no influence of sex on SSI incidence [14]. The impact of operative time and third molar extraction also remains controversial [9, 13, 14]. In the present study, we found no statistically significant associations between SSI incidence and patient background factors such as age, sex, and BMI; nor did we find statistically significant associations between SSI incidence and treatment-related factors such as operative time, blood loss, TAD placement, or third molar extraction. Although increasing the sample size might reveal potential associations, these variables are unlikely to represent major contributing factors to SSIs in this context.

Several reports from other countries have examined factors related to SSI in orthognathic surgery, particularly regarding the use of prophylactic antibiotics. Prior studies have explored both the types of antibiotics [3, 8, 13] and the duration of administration during the perioperative period [9, 11, 14–17]. One report has concluded that prolonged administration of penicillin, cefazolin/cephalexin, or amoxicillin-clavulanate reduces infection rates, and that short-term use of penicillin is associated with a higher infection rate [3]. Others have reported no significant difference between amoxicillin and amoxicillin-clavulanate[8], or between amoxicillin-clavulanate and cefuroxime[13], indicating that the optimal antibiotic regimen remains controversial. In our study, the ampicillin group showed a higher SSI rate, while the sulbactam/ampicillin group showed a lower rate, suggesting that oral commensal bacteria, including β-lactamase-producing strains, may have contributed to the infections. Among the 23 patients who developed postoperative SSI, bacterial cultures were performed in 6 cases, all of which detected α-streptococcus species. Although all bacterial isolates were susceptible to ampicillin in vitro, the higher SSI rate in the ampicillin group suggests that additional factors beyond antimicrobial resistance (such as patient-related conditions and perioperative wound management) may have influenced SSI occurrence.

Regarding the duration of antibiotic administration the literature remains divided: some advocate single-dose prophylaxis[11, 14, 15], while others support postoperative continuation, including prolonged regimens [9, 16–18]. In our study, each antibiotic group followed a standardized duration of administration, making direct comparisons of treatment length unfeasible. Further studies on optimal duration are warranted.

As described above, there is still limited consensus on perioperative antibiotic use in orthognathic surgery, and further accumulation of evidence is needed. In particular, data from Japan are scarce [9], and current Japanese guidelines are largely based on international literature [7, 8, 15, 19]. Given this context, our study, which focused exclusively on Japanese patients, offers important domestic evidence for perioperative antibiotic use in orthognathic surgery.

This study has several limitations. First, this was a single-center retrospective study, which may be subject to selection bias. Differences in surgical environments, instruments, and patient characteristics may have affected the outcomes. Moreover, unmeasured confounding factors, such as patients’ oral hygiene status or perioperative oral rinse protocols, may have influenced SSI incidence but were not fully accounted for in this analysis. In addition, patients who experienced a change in perioperative antibiotics were excluded; however, these changes were due to temporary drug shortages rather than infection-related issues or patient-specific risk factors. Therefore, we believe their exclusion did not systematically bias the results with respect to SSI risk. As antimicrobial susceptibility profiles (antibiograms) vary across regions and institutions, local monitoring and stewardship are essential for optimising infection control measures based on local data. Moreover, caution is warranted when extrapolating these findings to healthcare systems with different oral flora, resistance patterns, or perioperative protocols. Future prospective multicenter studies with systematic data collection and multivariate analyses are needed to overcome this limitation. Second, the sample size was limited and uneven across the four antibiotic groups. The ampicillin group was particularly small (*n*= 8), which reduces the reliability of the statistically significant difference observed compared to the sulbactam/ampicillin group and raises the possibility of a type I error. Although SSI incidence in the cefmetazole and clindamycin groups was lower than in the ampicillin group, these differences were not statistically significant, likely due to the small sample sizes. Given the limited number of SSI events and the small size of the ampicillin group, multivariable logistic regression was not feasible, and residual confounding cannot be excluded. A larger and more balanced sample would allow a more robust evaluation of optimal and alternative antibiotics in orthognathic surgery. A third limitation of this study is that the duration of antibiotic administration was not consistent across groups, reflecting institutional practice at the time. Patients in the clindamycin group received antibiotics for 4 postoperative days, whereas those in the other groups received them for 2 days. As mentioned above, although previous studies have reported conflicting findings regarding the optimal duration of prophylactic antibiotics in orthognathic surgery, this variation in administration period could represent a confounding factor[9, 11, 14–18]. Future prospective studies with standardized prophylactic regimens are warranted to clarify this issue. Finally, microbiological data were limited, as bacterial cultures were performed in only 6 of the 23 SSI cases. This was due to the absence of abscess formation or purulent discharge in the other cases. As a result, our findings regarding the likely causative pathogens and their resistance profiles should be interpreted with caution.

In 2015, the World Health Assembly adopted the Global Action Plan on Antimicrobial Resistance, highlighting the urgent need for antibiotic stewardship worldwide [20]. In 2017, the World Health Organization introduced the AWaRe classification as a framework to guide antibiotic use at global, national, and regional levels [21]. In our study, sulbactam/ampicillin, ampicillin, and clindamycin are categorized as Access agents, whereas cefmetazole is classified as a Watch agent. Our findings suggest that cefmetazole and clindamycin may serve as alternative options when sulbactam/ampicillin is unavailable. However, antibiotic selection for surgical prophylaxis must also consider factors beyond AWaRe, such as tissue penetration, surgical duration, and regional resistance patterns. Furthermore, caution remains warranted with clindamycin due to its association with pseudomembranous colitis [22, 23]. Taken together, while the AWaRe classification provides a valuable global framework for stewardship, further investigation is needed to refine prophylactic strategies in orthognathic surgery, including the appropriateness of sulbactam/ampicillin as a first-line agent and the optimal duration of administration.

## Conclusions

Our findings indicate that ampicillin monotherapy may be inadequate for SSI prevention in orthognathic surgery, whereas a combination with β-lactamase inhibitors such as sulbactam/ampicillin appears more effective. Furthermore, cefmetazole and clindamycin showed comparable efficacy in preventing infection, suggesting that they may be viable alternatives to sulbactam/ampicillin.

## Data Availability

The datasets used and/or analysed during the current study are available from the corresponding author on reasonable request.

## Abbreviations

BMI: Body mass index
BSSO: Bilateral sagittal split osteotomy
CDC: Centers for Disease Control and Prevention
SSI: Surgical site infection
TAD: Temporary anchorage device
